# Bacterial ageing in the absence of external stressors

**DOI:** 10.1098/rstb.2018.0442

**Published:** 2019-10-07

**Authors:** Urszula Łapińska, Georgina Glover, Pablo Capilla-Lasheras, Andrew J. Young, Stefano Pagliara

**Affiliations:** 1 Biosciences, University of Exeter, Exeter, Devon EX4 4QD, UK; 2 Living Systems Institute, University of Exeter, Exeter, Devon EX4 4QD, UK; 3 Centre for Ecology and Conservation, College of Life and Environmental Sciences, University of Exeter, Penryn Campus, Penryn, Cornwall TR10 9FE, UK

**Keywords:** ageing, protein aggregates, glucose uptake, single-cell analysis, senescence, bacteria

## Abstract

Evidence of ageing in the bacterium *Escherichia coli* was a landmark finding in senescence research, as it suggested that even organisms with morphologically symmetrical fission may have evolved strategies to permit damage accumulation. However, recent work has suggested that ageing is only detectable in this organism in the presence of extrinsic stressors, such as the fluorescent proteins and strong light sources typically used to excite them. Here we combine microfluidics with brightfield microscopy to provide evidence of ageing in *E. coli* in the absence of these stressors. We report (i) that the doubling time of the lineage of cells that consistently inherits the ‘maternal old pole’ progressively increases with successive rounds of cell division until it reaches an apparent asymptote, and (ii) that the parental cell divides asymmetrically, with the old pole daughter showing a longer doubling time and slower glucose accumulation than the new pole daughter. Notably, these patterns arise without the progressive accumulation or asymmetric partitioning of observable misfolded-protein aggregates, phenomena previously hypothesized to cause the ageing phenotype. Our findings suggest that ageing is part of the naturally occurring ecologically-relevant phenotype of this bacterium and highlight the importance of alternative mechanisms of damage accumulation in this context.

This article is part of a discussion meeting issue ‘Single cell ecology’.

## 1. Introduction

Ageing, or senescence, can be broadly defined as a decline in performance (reproductive success or survival) with advancing age. Ageing is now well documented across a diverse range of multicellular organisms [1,2], where it is thought to arise from the progressive accumulation of biomolecular damage and errors (hereafter collectively ‘defects’) in somatic cells with advancing organismal age [3–6]. It was once assumed that unicellular organisms do not age, as (in the absence of a germ/soma distinction) the progressive accumulation of biomolecular defects in a lineage of cells that divides symmetrically would ultimately lead to its extinction [7–10]. However, early research on unicellular organisms that exhibit morphologically *asymmetric* fission (e.g. the budding yeast *Saccharomyces cerevisiae* [11–14] and the bacterium *Caulobacter crescentus* [15]) revealed that the daughter cell inheriting the older structures of the parental cell (rather than the structures newly created during cell division) shows a longer doubling time (the time elapsed between its genesis and its division in to two daughter cells) than the original cell that produced it. Thus cells of these species show a progressive decline in reproductive potential with advancing age, meeting the definition of ageing [11–15]. These findings led to the suggestion that morphologically asymmetric fission could be a key condition for the evolution of ageing, as it allows cells to accumulate biomolecular defects (a strategy that can be favoured by selection when molecular error checking and/or damage prevention and repair entail costs; [10,16]) but avoid long-term lineage extinction by passing these defects more to one daughter cell than the other, via asymmetric fission [7–10].

More recently, however, work on the bacterium *Escherichia coli* suggests that cells of this species may also accumulate reproduction-retarding defects, and partition these asymmetrically between their two daughter cells, despite the absence of evident morphological asymmetry during cell division in this species [16–19]. These findings, coupled with similar findings from the fission yeast *Schizosaccharomyces pombe* [13], a eukaryotic species that also lacks clear morphological asymmetry during fission, suggest that morphologically asymmetric fission is not a necessary precondition for the evolution of ageing. Instead, as dealing with the accumulation of biomolecular defects is probably a universal challenge across the tree of life, in order to maintain viable clonal growth rates while enjoying the resource savings afforded by defect accumulation (i.e. ageing), early unicellular organisms, whether morphologically asymmetrical or not, may have evolved now-ancient mechanisms for the asymmetric passage of these defects to their daughter cells [20]. While several more recent studies have further strengthened the case for ageing in *E. coli* [21–24], a number of key uncertainties remain.

**Figure 1.**
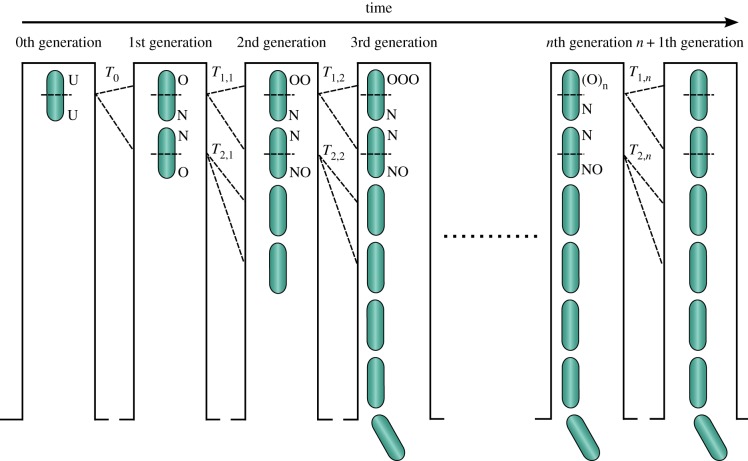
Schematics illustrating our approach for quantifying ageing in *E. coli*. After loading the mother machine with a stationary phase culture, we followed the bacterium at the top of each dead-end channel with unknown orientation (U) so that we could establish which one of its poles was the newer and which one the older pole. We defined *T*_0_ the time it took this bacterium to divide (not the actual doubling time of the cell as it last divided prior to being loaded in the mother machine). The two daughters of this bacterium inherit a new (N) and an old (O) pole each. We defined *T*_1,1_ and *T*_2,1_ as the doubling times of the first and second daughter from the top of the dead-end channel, respectively. Upon division the first daughter from the top gives rise to two further daughters: the daughter at the top inherits an OO and a N pole, the second daughter from the top inherits a N and a NO pole. After *n* generations, the first daughter from the top of the channel will have inherited (O)*_n_* and N poles and display a doubling time *T*_1,*n*_; the second daughter from the top of the channel will have inherited N and NO poles and display a doubling time *T*_2,*n*_. (Online version in colour.)

*Escherichia coli* cells are elongated rod-shaped structures with two ends or poles, one of which can be termed the ‘new pole’ (as it was newly constructed during the division of the parental cell; figure 1) and the other the ‘old pole’ (as it was inherited from the parental cell). Thus when any cell divides, one of its daughters (termed the ‘old pole daughter’) inherits the cell's old pole, while the other (termed the ‘new pole daughter’) inherits the cell's new pole (figure 1). By following seven consecutive bacterial divisions, Stewart *et al.* showed that the *E. coli* daughter cell receiving the old parental pole (the old pole daughter) grew more slowly than both the parental cell that produced it and the daughter cell receiving the new parental pole (the new pole daughter), thus providing the first compelling evidence of ageing in *E. coli* [19]. However, more recently, Wang *et al.* continuously tracked the growth rates of cells of three different *E. coli* strains over 200 generations of cell division and showed that the cells in the old pole lineage (the lineage of cells that continually inherits the parental old pole) actually showed no evidence of a progressive decline in growth rate between generations 10 and 200, contrary to what might be expected under continuous ageing [25]. Rang *et al* then reconciled these apparently conflicting findings by reanalysing one of the Wang *et al*. experiments and showed (i) that old pole daughters did have a longer doubling time than new pole daughters (i.e. that asymmetric fission was occurring), and (ii) that old pole daughters did have longer doubling times than their parental cell (i.e. that ageing was occurring) *if* the parental cell was itself a new pole daughter cell. Rang *et al.* hypothesized that the lack of continuous degeneration of cell growth rates within the old pole lineage in the Wang *et al.* study reflects an asymptotic state for the old pole lineage [21], in which accrued defects cease to further accumulate (an asymptote that may not yet have been reached in the original seven generation study by Stewart *et al.* [19]). Indeed, Rang *et al.* indicate that such an asymptotic state for the old pole lineage (which they term the ‘old pole attractor state’ [21]) had previously been predicted by a theoretical model of unicellular ageing [16]. Further examination of the temporal dynamic of cell doubling times in the old pole lineage over successive generations is therefore needed to clarify the generality of (i) the progressive ageing described by Stewart *et al.* [19], and (ii) the asymptotic state observed by Wang *et al.* [25] (and Rang *et al.* [21] in data from [25]).

In a further complication, a recent attempt to reconcile historically divergent findings on the existence of ageing in *E. coli* concluded that ageing only occurs in *E. coli* in the presence of ‘damage by an extrinsic agent’ [24]. Indeed, it was noted that all studies finding evidence of ageing in *E. coli* had used fluorescent proteins as a visual marker, while all studies failing to detect it had not [24]. This led to the suggestion that the ageing documented in these studies was induced by harmful effects of putative extrinsic damage agents, such as the strong light sources (lasers or LEDs) that were used to excite these proteins or the enforced expression of the fluorescent proteins themselves (which could conceivably increase the incidence of intra-cellular damage if their synthesis or processing reduced the effectiveness of the cell's damage mitigation and repair mechanisms; e.g. simply by using limited resources) [24]. Indeed, the enforced expression of any extrinsic protein could also retard reproduction via damage-*independent* mechanisms, simply by using resources that could otherwise have been used for growth. These observations could reflect one of two scenarios. First, ageing may be part of the ecologically-relevant natural phenotype of wild-type *E. coli*, if they encounter stressors with comparable physiological effects. Indeed, wider evidence supports the view that the detection and magnitude of ageing, both in unicellular and multicellular organisms, depends on the extent of organismal exposure to stressors [26–30]. Second, it is also conceivable that the ageing phenotype detected in *E. coli* is actually an aberrant artefact of the techniques employed to date to study it. Further attempts to characterize ageing in wild-type *E. coli* in the absence of fluorescent proteins and associated strong light sources could therefore shed light on the plausibility of these scenarios.

The biological mechanisms that give rise to the apparent ageing phenotype in *E. coli* remain poorly understood, and could involve a diverse array of pathways including oxidatively damaged proteins or misfolded protein aggregates [13,20]. Lindner *et al.* found that aggregates of misfolded proteins (imaged by fluorescently tagging the molecular chaperone IbpA) segregate asymmetrically upon cell division, passing differentially more to the old pole daughter than the new pole daughter, and accumulating over successive generations within the lineage of cells that consistently inherits the maternal old pole; a pattern consistent with a causal role for aggregates of misfolded proteins in yielding the ageing phenotype [17]. In a follow up study using the fluorescently tagged molecular chaperones DnaK, DnaJ, and ClpB, Winkler *et al.* showed that protein aggregates localized at the *E. coli* poles were asymmetrically segregated, whereas aggregates randomly distributed within the cell were symmetrically segregated between the daughter cells [31]. As both studies were conducted in the presence of the putative extrinsic damage agents that have been hypothesized to induce ageing in *E. coli* (fluorescent proteins and strong light sources; [24,26,27]), attempts to investigate the possible role of protein aggregates in *E. coli* ageing in the absence of such damage agents would now be worthwhile.

Here we seek to characterize ageing in wild-type *E. coli* BW21113 in the absence of the extrinsic damage agents recently hypothesized to cause it (fluorescent proteins and strong light sources; [24]) and investigate the role that misfolded-protein aggregates play in generating the ageing phenotype that we detect in this context. First, we use a microfluidic ‘mother machine’ device [25] coupled with time-lapse brightfield microscopy to study the doubling times of individual *E. coli* cells and their daughter lineages over up to 24 successive cell divisions. These wild-type cells do not express fluorescent proteins and we do not expose them to powerful light sources (see §2b). We test three specific predictions: (i) that old pole daughter cells will show longer doubling times than generation-paired new pole daughter cells (i.e. that asymmetric fission is occurring); (ii) that the lineage of cells that consistently inherits the old pole will show a progressive slowing in doubling times over successive generations (i.e. that ageing is occurring, as detected by Stewart *et al.* [19]); and (iii) that this progressive slowing of doubling times in the old pole lineage will eventually asymptote (reconciling the findings of Stewart *et al.* [19] and Wang *et al.* [25], as hypothesized by Rang *et al.* [21]). Second, we further characterize phenotypic asymmetry in generation-paired new and old pole daughter cells in this context by assessing their glucose accumulation profiles. We predicted that new pole daughter cells would show stronger glucose accumulation, if their shorter doubling times entail higher metabolic demands or if old pole daughter cells suffer pathological defects in their membrane transport capabilities. Third, we investigate the extent to which the observed asymmetry in doubling times of new and old pole daughter cells in this context can be attributed, as hypothesized, to the build-up and asymmetric partitioning among daughters of misfolded-protein aggregates (again in the absence of the extrinsic damage agents recently hypothesized to induce ageing).

## 2. Material and methods

### (a) Chemicals and cell culture

All chemicals were purchased from Fisher Scientific or Sigma-Aldrich unless otherwise stated. Lysogeny broth (LB) medium (10 g l^−1^ tryptone, 5 g l^−1^ yeast extract, and 0.5 g l^−1^ NaCl) and LB agar plates (LB with 15 g l^−1^ agar) were used for bulk bacterial growth. M9 minimal medium (7 g l^−1^ Na_2_HPO_4_, 3 g l^−1^ KH_2_PO_4_, 1 g l^−1^ NH_4_Cl, 0.5 g l^−1^ NaCl, 1 mM thiamine hydrochloride) was used for the ensemble and single cell investigation of amyloid aggregates of misfolded proteins. The wild-type *E. coli* BW25113 strain used to characterize ageing in the *absence* of fluorescent proteins and strong light sources was purchased from Dharmacon (GE Healthcare). A second *E. coli* BW25113 strain carrying a low copy number plasmid with the promoter region of the tryptophanase operon leader peptide *tnaC* inserted upstream of a gene for a fast-folding green fluorescent proteins (GFP) was also purchased from Dharmacon. An *E. coli* BL21 strain recombinantly expressing the amyloid Aβ peptide (M1-42) (UniProtKB ID P05067) was kindly provided by Dr Janet Kumita and Prof Sara Linse (University of Cambridge and Lund University, respectively). The latter two strains were used exclusively to confirm previous reports that *E. coli* expressing extrinsic proteins produces misfolded protein aggregates [17]. Overnight cultures of the three strains above were prepared by picking a single colony of *E. coli* from a streak plate and growing it in 100 ml fresh LB medium in a shaking incubator at 200 rpm and 37°C for 17 h.

### (b) Microfluidics and time-lapse microscopy for ageing phenotyping

In order to phenotype new and old pole daughter cells for (i) doubling times, (ii) glucose uptake and (iii) the existence of misfolded protein aggregates, we combined time-lapse microscopy with the microfluidic mother machine device whose fabrication and handling has been previously reported [25,32]. This device, made of polydimethylsiloxane (PDMS), is equipped with an array of dead-end microfluidic channels with width and height of 1.5 µm and a length of 25 µm. Because *E. coli* are rod shaped with a typical width and height of less than 1 µm and a length between 1 and 5 µm (depending on the specific strain, phase of growth and nutrient levels), each channel accommodates between one and 10 bacteria disposed in a single file (figure 1). This allows tracking of each single bacterium over time and, crucially for this work, distinguishing between old and new pole daughter bacteria because bacteria cannot exchange position within the single file. Of relevance for the current study, resource availability does not vary along the length of each channel of the mother machine [25]. In order to sustain bacterial growth, these channels are connected to a main microchamber (25 and 100 µm in height and width, respectively) continuously supplied with fresh nutrients (see section below). In this work the PDMS mother machine device was irreversibly sealed to a glass coverslip by exposing both surfaces to oxygen plasma treatment (10 s exposure to 30 W plasma power, Plasma etcher, Diener, Royal Oak, MI). Within 5 min after plasma treatment, the device was filled with 2 µl of a 50 mg ml^−1^ bovine serum albumin (BSA) solution and incubated at 37°C for 1 h. Meanwhile, spent LB broth and bacteria were prepared by centrifuging (10 min at 3000 g and 20°C) an overnight culture prepared as described above. The supernatant was filtered twice (Medical Millex-GS Filter, 0.22 µm, Millipore Corp.) and used to re-suspend the bacteria in their spent LB to an optical density of 75 at 595 nm. Then 2 µl of this highly concentrated bacterial suspension was injected in the main microchamber of the mother machine and individual bacteria allowed to diffuse in the lateral channels for 20 min. The mother machine was completed by the integration of fluorinated ethylene propylene inlet and outlet tubing (1/32″ × 0.008″) as previously reported [33]. The inlet tubing was connected to a flow-rate measuring device (Flow Unit S, Fluigent, Paris, France) controlling the pressure applied by a computerized pressure-based flow control system (MFCS-4C, Fluigent). The mother machine was mounted on an inverted microscope (IX73 Olympus, Tokyo, Japan) equipped with a 60×, 1.2 N.A. objective (UPLSAPO60XW, Olympus), and a sCMOS camera (Zyla 4.2, Andor, Belfast, UK). Brightfield illumination was provided via a halogen lamp (Olympus U-LH100 L-3) regulated at a beam power of 0.09 mW at the sample plane. For reference, when we image strains expressing GFP in fluorescence mode, we use a blue LED (CoolLED pE200white, maximal power = 200 mW, Andover, UK) with a power associated with the beam light of 7.93 mW at the sample plane (i.e. approximately 90 times higher power than the lighting under brightfield illumination). This equipment is controlled via a custom built Labview software to acquire a brightfield image (exposure time 0.03 s) of 10 mother machine channels (and the bacterial lineages that they contain; approximately 10–80 cells depending on the number of generations elapsed from initial seeding) every 2 min for the entire duration of each experiment. After acquiring the first image, the microfluidic environment was changed by flowing fresh LB at 100 µl h^−1^ for the entire duration of the experiment to sustain bacterial growth at 37°C.

### (c) Measurement of the doubling times of new and old pole daughter cells

By using the mother machine and time-lapse microscopy we tracked each individual bacterium and its progeny throughout each experiment. The doubling times *T*_1,*n*_ and *T*_2,*n*_ for the first and second cell from the top of each dead-end channel at the *n*th generation (the old and new pole daughter cells respectively, see figure 1 and §3a for further details) were measured as the lapses of time from the division of their parental cell at the (*n* − 1)th generation to each of their respective divisions. Bacterial division was assessed by eye through the images loaded in ImageJ and considered to have happened when two daughter cells became clearly distinguishable from their respective parental cell. In contrast to some other studies [21,34] we do not report data on the doubling times of the progeny of new pole daughters (the third and fourth bacteria from the top of the channel in figure 1) for two reasons. First, these cells very often left their channel before completing division, restricting the available sample sizes [33]. Second, as these cells were often close to the opening of the dead-end channel they could have experienced hydrostatic pressures that impacted their doubling times.

We performed two independent experiments seeding the mother machine with two different overnight *E. coli* cultures. In each experiment we measured the doubling times of 10 different pairs of old and new pole daughters held in 10 different dead-end channels. The cells were monitored for 11 generations in the first experiment ‘run A’ and 24 generations in the second ‘run B’.

### (d) Measurement of glucose uptake in new and old pole daughter cells

In order to measure the profile of glucose uptake kinetics in individual new and old pole daughter cells, we used the fluorescent glucose analogue 2-(*N*-(7-Nitrobenz-2-oxa-1,3-diazol-4-yl)Amino)-2-Deoxyglucose (2-NBDG) dissolved in M9 at 30 µM. *Escherichia coli* were grown in the mother machine for four generations before delivering 2-NBDG at 100 µl h^−1^ for 15 min. We acquired brightfield and fluorescence images (as described above) every minute using a fluorescein isothiocyanate (FITC) filter, a blue LED (see above) at 15% of its intensity, and an exposure time of 0.1 s.

Whole cell 2-NBDG fluorescence intensity for *n* = 40 pairs of old and new pole daughters (pooled together from three independent mother machines seeded with three different *E. coli* cultures) were extracted via a semi-automated approach based on ImageJ that was used to calculate the mean intensity in a rectangle drawn around the perimeter of individual bacteria. For each bacterium we estimated the fluorescence background as the mean intensity in a rectangle with the same dimensions and positioned at the same height (distance from the delivery chamber) in the next empty channel. This signal was subtracted from the corresponding single-bacterium fluorescence intensity.

### (e) Measurement of misfolded protein aggregates in new and old pole daughter cells

In order to quantify the formation of misfolded protein aggregates, we sought evidence of aggregate formation in bacterial inclusion bodies both via bright field imaging [17] and via fluorescence microscopy using the dye thioflavin T (ThT). This dye is widely employed to stain amyloid misfolded protein aggregates in both eukaryotic and bacterial species [35,36]. In order to optimize the staining protocol with ThT, we performed ensemble measurements by using a plate reader at 37°C (FLUOstar Omega, BMGLABTECH, Germany). In each of the 96-well non-binding plates (3881, Corning), we diluted ThT at different concentrations in 60 µl of growth medium (either LB or M9 minimal medium). We then added to each well 30 µl of an overnight *E. coli* culture and gently mixed with a pipette. We performed fluorescence measurements (excitation/emission: 430/480 nm) in triplicate for each of the following ThT concentrations: 0, 10, 20, 30, 40, and 50 µM. Our negative control, also performed in triplicate, constituted of bacteria-free 90 µl media at different ThT concentrations (0–50 µM). Fluorescence readings were performed every 4 min and the plate was shaken at 200 rpm for 130 s using double orbital setting before each measurement.

In order to characterize the levels of staining with ThT in individual bacteria, we injected ThT dissolved in M9 at 50 µM in the mother machine at 100 µl h^−1^ for 45 min. In order to investigate the effect of ageing on the formation of protein aggregates, we performed these measurements after growing *E. coli* for 24 generations in the mother machine. During incubation with ThT we acquired a brightfield image of 50 dead-end channels of the mother machine every 3 min. Upon acquiring each brightfield image the microscope was switched to fluorescent mode, ThT was excited by using a FITC filter and a blue LED (see above) at 20% of its intensity and a fluorescence image was acquired by using an exposure time of 0.03 s. These measurements were conducted during separate experimental mother machine runs from the runs that were used to characterize the ageing and asymmetric division phenotypes in §2b,c above (thereby avoiding the use of fluorescent proteins and strong light sources during the latter). For reference, these measurements were also carried out using the two strains expressing either GFP or amyloid Aβ peptides described in §2a.

We then used ImageJ to draw a 5-pixel wide line along the length of each bacterium and to extract both the brightness profile and the corresponding GFP or ThT staining profile for *n* = 10 pairs of old and new pole daughter cells from brightfield and fluorescence images, respectively. For each bacterium we then estimated a baseline in brightness (or fluorescence) as the average of the brightness (or fluorescence) of the 10 most central pixels of the bacterium, as misfolded protein aggregates are expected to accumulate in inclusion bodies located at the bacterial poles [17,37]. We then subtracted this brightness (or fluorescence) baseline from the bacterium brightness (or fluorescence) profile. We calculated the brightness (or fluorescence) of potential inclusion bodies *B_IB_* (or *F_IB_*) as the sum of at least three, thus excluding spurious image noise [38,39], spatially consecutive negative values (or positive values for fluorescence imaging) excluding the above mentioned 10 most central pixels of the bacterium.

### (f) Statistical analysis

Linear mixed models (LMMs) were employed to explain variation in doubling times of individual bacteria between generation 2 and 11. Polarity (old pole versus new pole daughter cell), generation number, experimental run (A or B), initial number of bacteria loaded per channel as well as the interaction between polarity and generation number were included as fixed effect predictors. Channel identity was added in the model as a random intercept effect (see the electronic supplementary material, table S1). Data from the experimental run B, from generation 2 to 24, were analysed including a similar set of fixed effect predictors, with the removal of the ‘run’ effect and the additional inclusion of a quadratic effect of generation number as well as its interaction with polarity (see the electronic supplementary material, table S2). The importance of fixed effect predictors was evaluated using nested likelihood-ratio test (LRT) until a minimum adequate model (MAM) was obtained, which retained every significant fixed effect (see the electronic supplementary material, tables S1 and S2). We report on the significance values of predictors retained in the MAM, removing the focal predictor and comparing the resulting simpler model to the MAM using an LRT. The significance values of predictors not included in the MAM were calculated by including the focal fixed effect back into the MAM and comparing the resulting more complex model against the MAM using an LRT. Model residuals were visually inspected and no important deviations from normality were found. Nor did we find evidence for model residual heteroscedasticity.

Statistical significance of the difference in glucose accumulation and misfolded protein aggregate formation between generation-paired old and new pole daughters was evaluated by a paired *t*-test. Statistical significance of the difference in misfolded protein aggregate formation between wild-type *E. coli* and GFP expressing or misfolded protein producing *E. coli* was evaluated via an unpaired *t*-test with Welch's correction.

## 3. Results

### (a) Asymmetry in doubling time between old pole and new pole daughters

In order to quantify ageing in *E. coli*, we measured the doubling times of individual bacteria and their progeny over several generations (11 for run A and 24 for run B). After loading the mother machine with stationary phase *E. coli*, we measured the time *T*_0_ that the bacterium at the top of the dead end of each channel took to divide (0th generation in figure 1; not the actual doubling time of the cell as it last divided prior to being loaded in the mother machine). While the initial number of bacteria loaded in to each channel varied from 1 to 10, this initial cell number did not affect *T*_0_ or the analysis presented below of the division dynamics of the top cell's daughter lineage (see the electronic supplementary material, tables S1 and S2). As the top cell in each channel had just been loaded in the mother machine, it was not clear at this stage which of its poles was the newer and which the older pole (we label both ‘unknown’ (U) in figure 1). Upon division, this top cell gave rise to two daughter cells, both inheriting an existing pole from the parental cell (now termed an old (O) pole in figure 1) and a new pole (N), just generated at the division septum. We then monitored both of these daughter cells until their division with doubling time *T*_1,1_ and *T*_2,1_ for the top and second daughter cell respectively in each dead-end channel (the second subscript index indicates the generation number). Because the original parental cell that divided at *T*_0_ was in an unknown orientation, these two daughter cells have an equal probability of being its old and new pole daughters. Accordingly, there was no significant difference between the doubling times *T*_1,1_ and *T*_2,1_ of these two cells (paired *t*-test: *t*_19_ = 0.538, *p* = 0.596, *n* = 20 daughter cell pairs from 10 channels in each of two runs).

When the first daughter at the top of the channel divided with doubling time *T*_1,1_, we could now be certain for the resulting generation of daughter cells (and all subsequent generations) that the top daughter cell produced in the channel was the old pole daughter cell (i.e. that it had inherited the parental cell's old pole (now denoted OO in this old pole daughter, having persisted for a generation; figure 1) as the parental cell's old pole must have been abutting the closed top end of the channel [25]). As a consequence we could now also be certain that the second daughter cell in the channel was the new pole daughter cell, having inherited the parental cell's new pole (now denoted NO in this new pole daughter, having persisted for a generation; figure 1). We denoted the doubling time of this top daughter cell *T*_1,2_ (and *T*_1,*n*_ in the *n*th generation as we tracked the old pole daughter cell lineage forward; figure 1) and the doubling time of the second daughter cell in the channel *T*_2,2_ (and *T*_2,*n*_ in the *n*th generation; figure 1). In the analyses below within §3a,b, we focus on the doubling times of these new pole daughter cells (*T*_2,*n*_ doubling times for *n* > 1) and old pole daughter cells (*T*_1,*n*_ doubling times for *n* > 1) produced by the lineage of cells successively inheriting the old parental pole.

**Figure 2.**
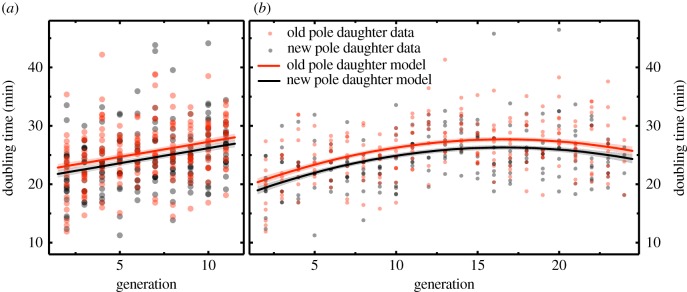
Ageing and asymmetric division in *E. coli.* Dependence of cell doubling time on generation number for old pole (red) and new pole daughter cells (black). (*a*) Old pole daughter cells took longer to divide than new pole daughter cells (LMM: $\chi_{1}^{2}=5.192$, *p* = 0.023; electronic supplementary material, table S1). For both cell types, division time progressively increased over successive generations (LMM: $\chi_{1}^{2}=38.327$, *p* < 0.001; electronic supplementary material, table S1). This analysis was carried out including data for generations 2–11 from both experimental runs (20 channels in total), controlling for variation among runs (see the electronic supplementary material, table S1 for full model description). (*b*) Inspecting the 24 generation dataset (run B) revealed that the progressive increase in division times gradually asymptotes to a steady state division time with increasing generation number (LMM: linear effect of generation number: $\chi_{1}^{2}=52.199$, *p* < 0.001; quadratic effect of generation number: $\chi_{1}^{2}=32.832$, *p* < 0.001; electronic supplementary material, table S2). Old pole daughter cells also took longer to divide than new pole daughter cells within this 24 generation dataset (LMM: $\chi_{1}^{2}=11.222$, *p* < 0.001; electronic supplementary material, table S2). Coloured lines and grey shaded area represent mean model predictions ± standard error (s.e.) for (*a*) the effect of generation number and polarity across experimental runs between generation 2 and 11 inclusive (electronic supplementary material, table S1), and (*b*) the effect of generation number and polarity for experimental run B between generation 2 and 24 inclusive (electronic supplementary material, table S2). Red and black dots illustrate raw data points for old pole and new pole daughter cells respectively, with darker point shading reflecting overlapping data points. (Online version in colour.)

Consistent with the expectation that old pole daughter cells inherit reproduction-retarding structures to a greater extent than new pole daughters, the doubling times of old pole daughter cells (*T*_1,*n*_ once *n* > 1) significantly exceeded those of new pole daughter cells (*T*_2,*n*_ once *n* > 1; LMM: $\chi_{1}^{2}=5.192$, *p* = 0.023; figure 2*a*; electronic supplementary material, table S1; see also figure 2*b*; electronic supplementary material, table S2), despite the absence of the ‘extrinsic damage agents’ hypothesized to yield the ageing phenotype [24]. This analysis was conducted using data from 10 channels monitored across each of the two experimental runs (i.e. 20 bacterial lineages in total). The dataset includes cell doubling times for generations 2–11 inclusive (we restricted the analysis to the generations over which both runs were monitored; see figure 2*b* and electronic supplementary material, table S2 for similar findings for the 24 generation run in isolation). The analyses simultaneously controlled for variation in doubling times owing to effects of the number of generations of cell division that had occurred (termed ‘generation number’; see below) and the identity of the focal experimental run (see the electronic supplementary material, tables S1 and S2 for full model outputs).

### (b) Progressive but asymptotic ageing of the old pole lineage

Note that as we track the old pole daughter lineage forward over *n* successive generations of cell division (figure 1), the older pole of the ‘old pole daughter cell’ (i.e. the cell at the top of the channel, when *n* > 1) will have persisted for longer and longer (denoted O*_n_* in the *n*th generation; figure 1), highlighting the possibility that this lineage progressively deteriorates with advancing generation number. Indeed, our analyses reveal that the doubling times of cells in the old pole lineage do significantly increase with increasing generation number (LMM: $\chi_{1}^{2}=38.327$, *p* < 0.001; controlling for effects of cell polarity (new versus old pole daughter cell), run and channel; electronic supplementary material, table S1); an effect that is readily apparent over the first 11 generations in both runs when inspecting the doubling time data (figure 2*a*). Inspection of the data from the 24 generation run (run B; figure 2*b*) suggests that this progressive increase in doubling times gradually asymptotes to a steady state doubling time with increasing generation number. Indeed, statistical analysis of the run B data in isolation (electronic supplementary material, table S2) confirms a significant positive effect of the linear term ‘generation number’ (LMM: $\chi_{1}^{2}=52.199$, *p* < 0.001; electronic supplementary material, table S2) and a significant negative effect of the square of generation number (i.e. its quadratic component; LMM: $\chi_{1}^{2}=32.832$, *p* < 0.001; electronic supplementary material, table S2), indicating that there is a progressive reduction in the gradient of the generation number effect with advancing generation number. While this quadratic model yields a best-fit curve which suggests that doubling times may start to *decrease* again beyond 16 generations (figure 2*b*), restricting the dataset to generations 17–24 inclusive reveals *no* significant effect of generation time on doubling time after generation 16 (LMM: $\chi_{1}^{2}=0.757$, *p* = 0.384). These findings are consistent with an asymptote in the doubling times of this lineage of cells that consistently inherits the parental old pole. There was no statistically significant change in the magnitude of the difference in doubling times between generation-paired old and new pole daughter cells with increasing generation number, either when the first 11 generations are combined together for both runs (LMM polarity × generation number interaction: LMM, $\chi_{1}^{2}=0.001$, *p* = 0.971; electronic supplementary material, table S1) or in the 24 generation dataset in isolation (LMM polarity × generation number interaction: LMM, $\chi_{1}^{2}=0.545$, *p* = 0.460, polarity × generation number interaction: LMM, $\chi_{1}^{2}=0.459$, *p* = 0.498; electronic supplementary material, table S2).

### (c) New pole daughters accumulate glucose more quickly than old pole daughters

**Figure 3.**
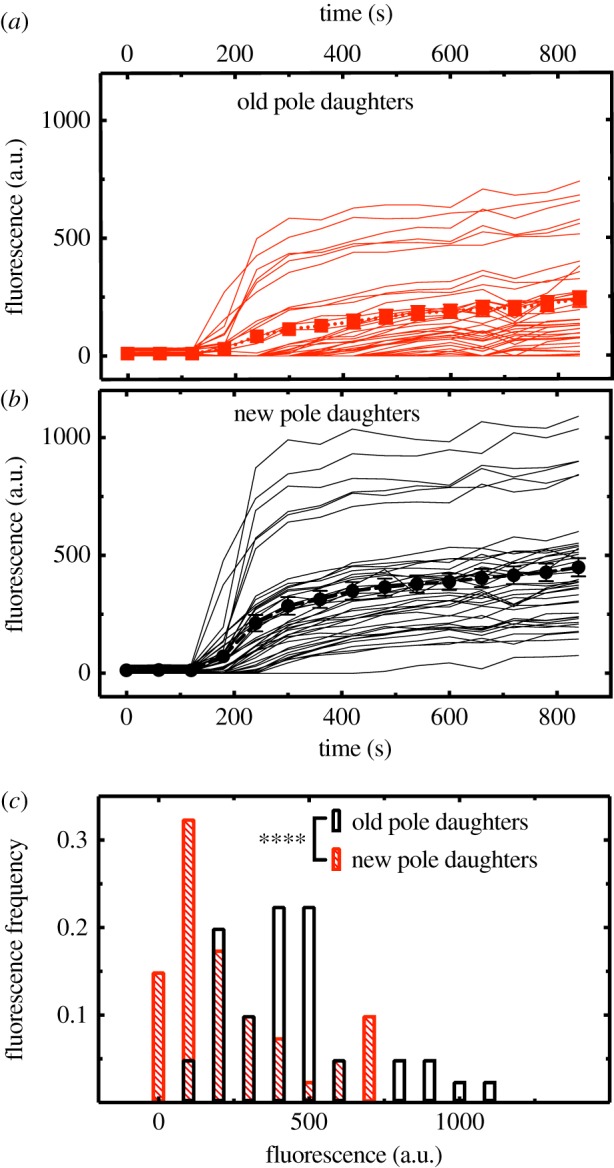
New and old pole daughters differ in their glucose uptake patterns. (*a*) Uptake of the fluorescent glucose analogue 2-(*N*-(7-Nitrobenz-2-oxa-1,3-diazol-4-yl)Amino)-2-Deoxyglucose (2-NBDG) in 40 pairs of old pole and (*b*) generation-matched new pole daughter cells collated from three independent mother machine experiments. Each pair was hosted in a different dead-end channel of the mother machine and constituted the 4th generation grown in the mother machine from an *E. coli* overnight culture. Squares and circles (and error bars) represent the means (and standard errors) of the single-cell uptake traces. 2-NBDG was introduced in the mother machine at *t* = 0 s. (*c*) At the end of each experiment (*t* = 840 s) new pole daughters (black empty bars) had accumulated intracellular glucose to a significantly higher level than old pole daughters (red filled bars; paired *t*-test, *n* = 40 pairs, *p* < 0.0001). (Online version in colour.)

To investigate whether the slower doubling times of old pole than new pole daughter cells were associated with correlated differences in their resource accumulation patterns, we measured the accumulation of the fluorescent glucose analogue 2-NBDG in individual bacteria within the mother machine. 2-NBDG has been widely employed to study the dynamics of glucose uptake both at the population and single-cell level [40,41]. After growing *E. coli* in the mother machine for four generations, we quantitatively compared the glucose accumulation kinetics of old pole daughter cells (figure 3*a*) and generation-matched new pole daughters (figure 3*b*). Inspection of the data reveals that new pole daughter cells accumulated 2-NBDG more quickly. Indeed, despite a high degree of heterogeneity in the final levels of 2-NBDG accumulation, after 14 min (840 s) of incubation with 2-NBDG new pole daughters had accumulated a significantly larger amount than generation-paired old pole daughters (*n* = 40 pairs, *t*_39_ = 11.3, *p* < 0.0001; figure 3*c*). As we and others [25] have performed separate experiments showing that 2-NBDG uptake is not affected solely by the position of individual bacteria in the microfluidic channels (electronic supplementary material, figure S1; see also [25]), the above glucose uptake contrasts between new and old pole daughters cannot be readily attributed to channel-position effects alone.

### (d) Asymmetry in doubling time does not correlate with the asymmetric inheritance of protein aggregates

**Figure 4.**
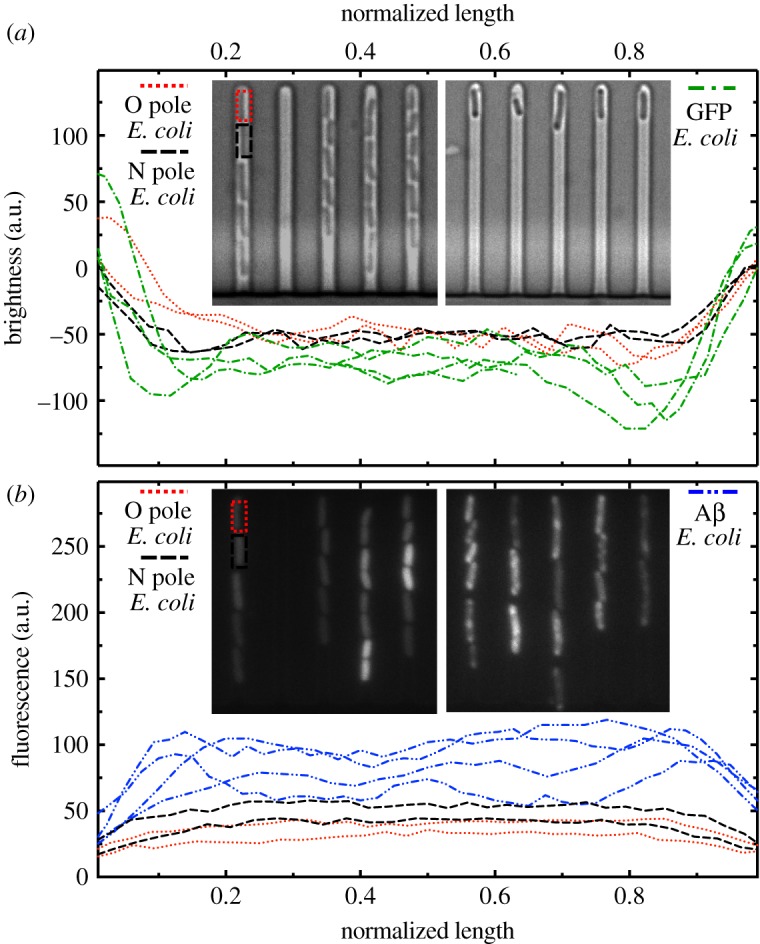
Ageing does not correlate with the formation of misfolded protein aggregates. (*a*) Brightness profile along the length of two pairs of old and new pole daughters at the 24th generation of growth in a mother machine seeded with an overnight *E. coli* culture (red dotted and black dashed lines, respectively) and of individual *E. coli* expressing exogenous GFP and loaded in a different mother machine from a different overnight culture (green dotted dashed lines). Only the latter exhibit significant depth in brightness in proximity of the bacterial poles resembling previously reported misfolded protein aggregates [16]. Formal statistical comparisons of brightness profiles (see §2e) showed that: (i) the wild-type *E. coli* cells displayed significantly less evidence of pole-associated dark foci than *E. coli* expressing exogenous GFP (*n* = 30 cells; *p*-value = 0.005); and (ii) wild-type *E. coli* old pole daughters did not show significantly stronger evidence of pole-associated dark foci than same-generation new pole daughters (a paired *t*-test of *n* = 10 pairs returned a *p*-value = 0.4). (*b*) ThT fluorescence profile along the length of two pairs of old and new pole daughters at the 24th generation of growth in a mother machine seeded with an overnight *E. coli* culture (red dotted and black dashed lines, respectively) and of individual *E. coli* expressing exogenous misfolded proteins after 4 h induction with IPTG dissolved in LB (to stimulate amyloid beta 42 production) in the mother machine from an overnight culture (blue dashed double dotted lines). Only the latter exhibit significant peaks in ThT fluorescence in proximity of the bacterial poles. Formal statistical comparisons of ThT fluorescence profiles (see §2e) revealed that: (i) the wild-type *E. coli* cells showed significantly less evidence of pole-associated ThT staining than *E. coli* expressing exogenous misfolded protein aggregates (*n* = 30 cells; *p*-value = 0.002); and (ii) wild-type *E. coli* old pole daughters did not show significantly stronger evidence of pole-associated ThT staining than same-generation new pole daughters (a paired *t*-test of *n* = 10 pairs returned a *p*-value = 0.2). (Online version in colour.)

A number of reports have suggested the possible accumulation of aggregates of misfolded proteins near old bacterial poles [17,31]. These aggregates are often sequestered in dedicated bacterial compartments, known as inclusion bodies, which can be observed as dark foci in brightfield or phase contrast images (e.g. see right inset in figure 4*a*) [17]. It has therefore been hypothesized that: (i) the asymmetry in the doubling times of new and old pole daughter cells arises from asymmetries in the inheritance of protein aggregates [17,18], and (ii) the progressive increase in doubling times within the old pole lineage arises from the progressive accumulation of protein aggregates in this lineage [19]. We therefore sought to establish whether protein aggregates occur with higher incidence in old than new pole daughters and whether they accumulate over successive cell divisions within the old pole lineage. We employed two approaches: first we used brightfield imaging to seek evidence of dark foci betraying the presence of inclusion bodies [17], and second we externally administered the dye ThT to stain for misfolded protein aggregates *in vivo* [36].

Our brightfield imaging of wild-type *E. coli* revealed no microscopic evidence of inclusion body formation (dark foci) throughout the 24 consecutive generations of cell division. Instead, both old and new pole daughters exhibited a uniform brightness profile across the length of the cell (red dotted and black dashed lines, respectively, in figure 4*a* and corresponding inset). Moreover, statistical comparisons of brightness profiles (see §2e) confirmed that after 24 generations of cell division: (i) the wild-type *E. coli* cells showed significantly less evidence of pole-associated dark foci than *E. coli* cells expressing GFP, which has been shown to trigger protein aggregate formation [37] (green dashed dotted lines in figure 4*a*; *n* = 30 cells, *t*_10_ = 3.6, *p* = 0.005); and (ii) wild-type *E. coli* old pole daughters did not show significantly stronger evidence of pole-associated dark foci than same-generation new pole daughters (*n* = 10 pairs of cells, *t*_9_ = 0.9, *p* = 0.4).

We corroborated these findings by introducing in the mother machine the dye ThT, that is commonly employed to stain misfolded protein aggregates *in vitro* [42]. First, we optimized the experimental parameters by performing plate reader measurements to show that ThT does stain *E. coli* populations growing in LB or M9 media (electronic supplementary material, figure S2a and S2c, respectively). Because without the addition of *E. coli*, we recorded a much stronger background signal in the presence of LB compared to M9 (electronic supplementary material, figure S2b and S2d, respectively), we decided to use M9 as the solvent for ThT when subsequently pulsing the mother machine with ThT for the purposes of single-cell characterizations. We then validated this approach for staining protein aggregates in individual live bacteria in the mother machine by using an *E. coli* strain bearing an inducible plasmid containing the *Aβ(M1-42)* gene encoding the peptide amyloid beta 42 that readily accumulates in the inclusion bodies of *E. coli* [43]. Indeed, when we stained these cells with ThT after 4 h induction with isopropyl β-D-1-thiogalactopyranoside (IPTG) dissolved in LB (to stimulate amyloid beta 42 production), we measured a strong fluorescence signal from the bacterial poles (blue dashed double dotted lines and right inset in figure 4*b*). The fluorescence signal co-localized with the dark foci observed in the corresponding brightfield images, lending strength to the view that dark foci observed in brightfield can be used as a proxy for the quantification of protein aggregate formation [44].

We then used this ThT staining approach to achieve single-cell protein aggregate characterizations for wild-type *E. coli* within the mother machine, to establish whether after 24 generations of cell division (i) pole-associated ThT fluorescence is apparent, and (ii) old pole daughter cells show stronger evidence of pole-associated ThT fluorescence. In accordance with our brightfield findings above, we did not find evidence of ThT fluorescence localization at the bacterial poles at generation 24. Instead, we observed a uniform intracellular staining with ThT for both old and new pole daughter cells (red dotted and black dashed lines, respectively, and left inset in figure 4*b*). This was possibly owing to the binding of ThT to distributed misfolded proteins within the cell or to other bacterial macromolecules such as total RNA or genomic DNA [45]. Statistical comparisons of ThT fluorescence profiles (see §2e) confirmed that after 24 generations of cell division (i) the wild-type *E. coli* cells showed significantly less evidence of pole-associated ThT staining than *E. coli* cells expressing the peptide amyloid beta 42 (*n* = 30 cells, *t*_9_ = 4.5, *p* = 0.002), and (ii) wild-type *E. coli* old pole daughters did not show significantly stronger evidence of pole-associated ThT staining than same-generation new pole daughters (*n* = 10 pairs of cells, *t*_9_ = 1.4, *p* = 0.2).

Taken together these observations suggest that the asymmetric doubling times of new and old pole daughters, and the progressive slowing of doubling times in the old pole lineage, reported above for wild-type *E. coli* do not correlate with the incidence of readily detectable misfolded protein aggregates.

## 4. Discussion

We sought to characterize ageing in wild-type *E. coli* in the absence of the extrinsic damage agents recently hypothesized to cause it [24], and to investigate the role that misfolded-protein aggregates may play in generating the ageing phenotype. Crucially, our findings reveal evidence of: (i) a progressive increase in the doubling times of the cells that consistently inherit the maternal old pole, over the course of the first 11 generations of cell division (figure 2*a*); and (ii) a persistent deficit in the doubling times of old pole daughters relative to generation-matched new pole daughters (figure 2*a*,*b*). These findings corroborate the key findings of Stewart *et al.* and Lindner *et al.* [17,19] in suggesting that *E. coli* do indeed age and that this may arise in part via functionally asymmetric cell division, despite the absence of evident morphologically asymmetric division in this organism. Our study extends those findings by providing evidence that both phenomena occur in wild-type *E. coli* in the *absence* of the extrinsic damage agents recently hypothesized to cause them [24]. Together these findings lend strength to the view that ageing is part of the ecologically-relevant natural phenotype of wild-type *E. coli*, rather than an aberrant artefact of the techniques employed to date to study it. Our findings also highlight complexity in the ageing phenotype of this organism, by confirming an apparently asymptotic trajectory of reproductive ageing (reconciling the findings of Stewart *et al.* [19] and Wang *et al.* [25], as hypothesized by Rang *et al.* [21]) and by questioning the hypothesized role of misfolded protein aggregates in generating the ageing phenotype. We discuss these findings in turn below.

While the doubling times of the old pole daughter lineage progressively increased over the first 11 generations of cell division (figure 2*a*), monitoring the lineage over 24 generations revealed formal statistical evidence that this rate of increase slows, consistent with an asymptotic trajectory towards a steady state doubling time (figure 2*b*). This trajectory illustrates how the findings of Stewart *et al.* (a progressive decrease in cellular growth rate within the old pole lineage over the first seven generations of cell division; [19]) can be reconciled with those of Wang *et al.* (no progressive decrease in cellular growth rate between generations 10 and 200; [25]), as hypothesized by Rang *et al.* [21]. This asymptotic pattern suggests that the lineage of cells which continually inherits the maternal old pole actually ceases to show evidence of (further) reproductive ageing beyond a threshold number of cell divisions. This behaviour is somewhat enigmatic as to the extent that the observed slowing of doubling times reflects the accumulation of biomolecular defects, this finding suggests that the old pole lineage may cease to further accumulate defects beyond a certain point, and it is not immediately apparent why this should be the case. While our observations of such an asymptote (and the steady state old pole lineage growth rates observed by Wang *et al.* [25]) are consistent with the suggestion that an ‘old pole attractor state’ exists beyond which the cells of the old pole lineage cease to accumulate damage [16,21], in the absence of a knowledge of the underlying mechanisms it remains unclear whether the core rationale of the theoretical model that predicted this asymptotic state [16,21] captures the processes at play in reality. Indeed, suggestions that this asymptotic state reflects a cessation of progressive damage accumulation in the old pole lineage would seem inconsistent with a key second observation in Wang *et al.*'s work: that after 50 generations of cell division the old pole lineage of cells shows evidence of a progressive decrease in survival probability (despite maintaining constant growth rates). This latter finding led to the suggestion that the old lineage progressively accumulates a lethal factor that ultimately reduces survival probability in the absence of effects on growth [25]; the old pole lineage in our study might therefore have continued to accumulate biomolecular defects beyond the apparent doubling time asymptote observed here, with implications downstream for cellular survival and/or reproduction. That said, as Wang *et al.*'s work was carried out using the putative extrinsic damage agents discussed above, it would seem conceivable that the ‘age-related’ declines in survival so documented could themselves be an artefact of the use of fluorescent proteins and the strong light sources used to excite their fluorescence [46].

Another conceivable explanation for the apparent asymptote in reproductive ageing within the old pole lineage, is that the initial progressive slowing in division times is actually an artefact of the starting conditions of the experiment. As the mother machine was initially loaded with bacteria from an overnight culture, it is possible, for example, that the early dynamics in our experimental runs were impacted in part by the bacteria transitioning from stationary phase to exponential growth and/or acclimation to the mother machine environment. However, it seems unlikely that these processes alone can account for the initial division time dynamics in our study for several reasons. First, the transition from stationary phase to exponential growth would be expected to be associated with a progressive decrease in cell division times, rather than the progressive increase that we documented within the old pole lineage. Indeed, the first cells loaded in to the mother machine, whose polar orientation was unknown, leading to the discarding of their division times from further analysis (*T*_0_; figure 1), showed much slower times to first division than subsequent generations of cells (*T*_0_ = 72 ± 3 min and *T*_1,1_ = 24 ± 1 min calculated as the means and standard error of the measurements in runs A and B). So in our study an immediate decrease in division times (consistent with the emergence from stationary phase) was then followed by a progressive increase in division times over multiple generations, consistent with the expectation of ageing in the old pole lineage. Furthermore, this progressive increase in division times persisted for around 11 generations and thus for over 5 h continuous exposure to fresh nutrients, whereas major gene expression profile remodelling during emergence from stationary phase occurs within 2 h of exposure to fresh nutrients [47]. Finally, the measured progressive increase in division times in our study mirrors the patterns originally reported for cellular growth rates within the old pole lineage by Stewart *et al*. [19], whose experiments were initiated using bacteria in exponential rather than stationary phase and were not performed in the mother machine. Acclimation to the mother machine environment might also be expected to yield an initial decrease in cell division times rather than the progressive increase observed, but the latter pattern would seem conceivable if, for example, the bacteria plastically adjusted their growth (i) in response to a changing social environment (e.g. as cells accumulate in the channel with successive rounds of division), or (ii) in response to being physically constrained in a microfluidic channel. However, our findings suggest that initial variation in the number of bacteria loaded in to each channel did not impact the division times of the top cell in the channel (see the electronic supplementary material, tables S1 and S2), and the number of cells within the channel (which can only house eight cells) will also have been largely invariant following three generations of cell division, yet the progressive division time dynamic persists for around 11 generations. It also seems unlikely that the physical constraints of the microfluidic channel or acclimation to the mother machine can account for our findings, as our findings qualitatively match the growth dynamics observed in Stewart *et al*. [19], in whose experiments bacteria were free to grow in two dimensional space. That said, the apparent transience of reproductive ageing within the old pole cell lineage in *E. coli* does highlight a need for closer attention to potential effects of initial conditions in future work on ageing in this organism.

Our findings revealed strong evidence of asymmetric division in *E. coli*, with new pole daughter cells showing significantly faster doubling times and markedly faster glucose accumulation rates than generation-matched old pole daughters. These findings cannot be readily attributed to old pole daughters having poorer access to nutrients because they occupy the very end of the dead-end channel (while generation-matched new pole daughters were consistently one cell-length closer to the source of the nutrients at the open end of the channel), for several reasons. First, the rate of diffusion of nutrients along the dead-end channel has been found to be two orders of magnitude higher than the rate of uptake of nutrients by the second bacterium from the top of the channel (the position occupied by the new pole daughters that we studied), such that nutrient uptake by the new pole daughter should not appreciably impact nutrient availability for the old pole daughter. This was demonstrated by Wang *et al.* in their seminal work introducing the mother machine, by imaging and measuring both the diffusion and uptake of nutrients (fig. S6 in [25]), leading them to conclude that (i) all bacteria in the channel are under a single and constant level of nutrient supply, and (ii) there is no accumulation of bacterial waste products in the channel. Second, as a further control in this study we loaded the mother machine with an aliquot of *E. coli* taken from an overnight culture and measured the uptake of the fluorescent glucose analogue 2-NBDG in the first and second bacterium from the top of 30 dead-end channels that each contained two bacteria. As these bacteria had been randomly drawn from the original culture and had yet to divide in the mother machine, they were of an unknown pole age and were in an unknown orientation. Our measurements show that the uptake of 2-NBDG was *not* significantly different between pairs of the first and second bacteria from the top of the channel in this context (electronic supplementary material, figure S1; unlike the generation-matched old and new pole daughters monitored in these same channel locations; figure 3), supporting the conclusion of *Wang et al.* that all the bacteria in the channel experience a comparable level of nutrient supply. Third, there was no significant difference in the doubling times of the first (*T*_1,1_) and second (*T*_2,1_) cells from the top of the channel following the first division of the original top cell loaded in to the mother machine (see results). As these two cells have an equal probability of being the old and new pole daughters of the original top cell, this analysis strongly suggests that it is the cells' pole age *per se*, rather than their position within the channel, that yields the doubling time differences documented here between new and old pole daughters.

The consistently slower division times of old than generation-matched new pole daughter cells, documented here in wild-type *E. coli* in the absence of fluorescent proteins (see also [34,48] for evidence to this effect), corroborate previous findings obtained in the presence of such putative ‘extrinsic damage agents’ [17,19,21,31]. As such, these findings lend strength to the view that unicellular organisms, whether they show apparent morphological asymmetry or not, may partition their cell components (potentially including accumulated biomolecular defects) asymmetrically between their daughter cells [12,13,15,20,49,50]. Selection may have favoured such a strategy if it affords the organism the resource savings that may arise from allowing biomolecular defects to accumulate, while averting the lineage extinction that would otherwise be expected to arise from symmetrically allocating such accumulated defects between daughter cells [10,16,17,19,21,31]. While it is certainly possible that the accumulation and partitioning of biomolecular defects play a central role in generating the performance asymmetry between new and old pole daughter cells, attention should also be paid to alternative explanations, given the limited understanding to date of the underlying mechanisms (see also discussion below). Notably, it is possible that the performance asymmetry reflects the asymmetric partitioning of essential bacterial components, rather than detrimental biomolecular defects, between new and old pole daughter cells. For example, it is known that ribosomes preferentially localize at the bacterial poles [51], thus costs or constraints associated with its partitioning between daughter cells could conceivably lead to its differential inheritance by new pole daughters. Were this the case, positive associations between ribosomal availability and cellular growth rates [52] could leave new pole daughter cells at an initial growth rate advantage. Indeed, it would seem plausible that asymmetric division in *E. coli* evolved in part as a response to costs entailed in achieving the symmetrical partitioning of cell components that are not uniformly distributed throughout the cell (and/or absolute constraints on a cell's ability to achieve this [48]), rather than primarily as a strategy to asymmetrically partition accumulated biomolecular defects [7–10,16].

Our glucose uptake measurements in paired old and new pole daughters demonstrate, for the first time to our knowledge, that the latter accumulate glucose at higher intracellular levels than the former. This finding suggests that the asymmetric doubling times of new and old pole daughters described above are accompanied, as one might expect, by differences in the biochemical makeup of the two types of cell. The difference in glucose accumulation detected here could reflect a range of mechanistic differences between the two groups that cannot be teased apart at this stage. First, new pole daughters may take up glucose at higher rates in order to support greater nutrient requirements associated with their shorter doubling times. Second, the glucose accumulation contrast could instead reflect differences in the membrane transport pathways of new and old pole daughters [50,53]. For example, Bergmiller *et al.* found that biased partitioning across daughter cells of the multidrug efflux pump TolC-AcrAB was responsible for lower accumulation of xenobiotics in old pole daughters, which conferred them higher antibiotic tolerance [50]. Indeed, differentially effective efflux pathways or deficits in influx pathways could each conceivably play a role in the reduced glucose accumulation phenotype observed here among old pole daughters. Third, as 2-NBDG is readily decomposed and used upon uptake [54], it is also conceivable that new and old pole daughters uptake glucose (2-NBDG) at comparable rates, but old pole daughters actually metabolize that glucose more quickly (e.g. to overcome biomolecular defects), leading to a lower net rate of accumulation of the substrate. Further work investigating the relative roles of differences in transport pathways versus metabolic demands and utilization rates in precipitating this difference in glucose accumulation between new and old pole daughters would now prove valuable.

Finally, we did not find evidence for the formation of misfolded-protein aggregates within the old pole lineage, either when seeking evidence of dark foci under brightfield or via the staining of amyloid misfolded-protein aggregates using the dye ThT. Specifically, our investigations revealed no evidence that misfolded protein aggregates (i) progressively accumulate within the old pole lineage, or (ii) are asymmetrically partitioned between (or apparent in) old and new pole daughters. These findings suggest that, when studied without fluorescent proteins and strong light sources, the ageing phenotype in *E. coli* can manifest in the absence of readily detectable misfolded protein aggregates. Notably, our findings contrast with those of Lindner *et al.* [17] and Winkler *et al.* [31], which showed that the old pole daughter lineage accumulates misfolded protein aggregates in pole-associated inclusion bodies that are asymmetrically inherited by old and new pole daughters. One potential explanation for this disparity is that both of the latter studies were conducted using *E. coli* strains modified to produce fluorescent proteins (in order to localize and quantitate misfolded protein aggregates) [24,26,27]. As the heterologous expression of such proteins in *E. coli* can lead to higher levels of misfolded protein aggregation [37], it would seem conceivable that the extent of aggregation previously observed could be a product in part of the techniques used to study it. Another potential explanation for this disparity, however, is that misfolded protein aggregates were indeed present in our study, but the techniques that we employed to study them actually failed to detect them. This seems unlikely, however, as misfolded protein aggregates are often conspicuous as dark foci under brightfield microscopy [17,44], and amyloid misfolded protein aggregates have been documented within bacterial inclusion bodies [37] and are known to be stained by the dye ThT [36]. That said, it is conceivable that unstressed and naturally ageing wild-type bacteria produce protein aggregates that (i) are not apparent as dark foci under brightfield (e.g. they could be too small), *and* (ii) are of an exclusively non-amyloid form that might therefore not be stained by ThT. Under these circumstances our combined approaches could indeed have failed to detect an active role for protein aggregates. Either way, our findings highlight the importance of attention to alternative mechanisms of defect accumulation and partitioning in unicellular organisms, particularly given the general expectation that multiple mechanisms of deterioration are likely to act in concert to generate ageing phenotypes [3,6,17,55]. Future work in this context might usefully focus on the role of the defect accumulation mechanisms implicated in the ageing of other organisms [20], such as oxidatively-damaged proteins [13].

## 5. Conclusion

Taken together, our findings provide evidence of ageing in wild-type *E. coli* in the absence of the extrinsic stressors recently hypothesized to cause it [24]. These findings lend strength to the view that ageing is indeed part of the naturally occurring ecologically relevant phenotype of this bacterium, rather than an artefact of the techniques used to date to study it. That said, our work does also highlight enigmatic aspects of the ageing phenotype of *E. coli* that warrant closer attention; most notably the existence of an apparent asymptote in reproductive ageing within the old pole lineage, the evolutionary and mechanistic origins of which remain poorly understood. Our findings also question a central role for misfolded protein aggregates as the reproduction-retarding structure responsible for the ageing phenotype in this organism [17,31], highlighting the potential importance of alternative mechanisms for biomolecular defect accumulation (at least when ageing is studied in the absence of fluorescent proteins). As *E. coli* undergoes morphologically symmetrical division, our findings also lend strength to the view that an apparent morphological asymmetry in cell structure is not a necessary precondition for the evolution of ageing [19]. Instead, unicellular organisms may have evolved now-ancient mechanisms for the asymmetric partitioning of accumulated defects between their daughter cells, as a strategy to deal with the universal challenge of biomolecular defect accumulation [12,13,18–20,49].

## Supplementary Material

Supplementary Material

## Data accessibility

The datasets supporting this article will be uploaded on Figshare.
