# Supplementary material for: Bacterial ageing in the absence of external stressors
Source: Philos Trans R Soc Lond B Biol Sci. 2019 Oct 7;374(1786):20180442. doi: 10.1098/rstb.2018.0442 (PMC6792439; doi:10.1098/rstb.2018.0442)

Bacterial ageing in the absence of external stressors

Ursula Lapinska,^1,2^ Georgina Glover,^1,2^ Pablo Capilla-Lasheras,^3^ Andrew J. Young,^3^ Stefano Pagliara^1,2^

^1^Biosciences, University of Exeter, Exeter, Devon EX4 4QD, UK

^2^Living Systems Institute, University of Exeter, Exeter, Devon EX4 4QD, UK

^3^Centre for Ecology and Conservation, College of Life and Environmental Sciences, University of Exeter,

Penryn Campus, Cornwall TR10 9FE, UK

**Keywords:** microfluidics; ageing; protein aggregates; glucose uptake; single-cell analysis; senescence; bacteria; *E. coli*

*Authors for correspondence ([s.pagliara@exeter.ac.uk](mailto:s.pagliara@exeter.ac.uk); [a.j.young@exeter.ac.uk](mailto:a.j.young@exeter.ac.uk); u.lapinska@exeter.ac.uk).

Supplementary Material

**Table S1.** (A) Likelihood-ratio test results and (B) effect size estimates from the minimum adequate model (MAM) explaining variation in bacterium doubling time (min) between generations 2 and 11 inclusive (we combine data from both experimental runs A and B, fitting ‘Run’ as a fixed effect predictor). The MAM contains only those predictors that explained significant variation in cell doubling times; in this case Polarity (new pole daughter cells showed significantly shorter doubling times than old pole daughter cells), Generation number (there was a progressive increase in the doubling times of cells with each successive generation of cell division), and Run (the doubling times of the cells in experimental run B were shorter than those in run A). There was no evidence that the effect of Polarity (new vs old pole daughter) changed with increasing generation number (i.e. there was no significant Polarity x Generation number interaction), and the initial number of bacteria in the run had no effect on the division times of the focal cells.

| **A) Likelihood-ratio test results** |  |  |  |
| --- | --- | --- | --- |
| **Predictor** | **χ^2^** | **df** | **p** |
| Polarity | 5.192 | 1 | 0.023 |
| Generation number | 38.327 | 1 | < 0.001 |
| Run | 30.277 | 1 | < 0.001 |
| Polarity x Generation number | 0.001 | 1 | 0.971 |
| Initial number of bacteria | 0.068 | 1 | 0.794 |
|  |  |  |  |
| **B) Minimum adequate model estimates** |  |  |  |
| **Fixed effects** | **Estimate** | **SE** | |
| Intercept | 23.893 | 0.677 | |
| Polarity (new pole daughter effect) | -1.084 | 0.476 | |
| Generation number | 0.517 | 0.082 | |
| Run (Run B effect) | -3.588 | 0.476 | |
|  | **Variance** | | |
| **Random effects**: Channel | ~ 0 | | |
| **Residual variance** | 22.01 | | |

**Table S2.** (A) Likelihood-ratio test results and (B) effect size estimates from the minimum adequate model (MAM) explaining variation in bacterium doubling time (min) between generations 2 and 24 inclusive for experimental run B. The MAM contains only those predictors that explained significant variation in cell doubling times; in this case Polarity (new pole daughter cells showed significantly shorter doubling times than old pole daughter cells) and Generation number (there was a progressive increase in the doubling times of cells with each successive generation of cell division – i.e. positive Generation number^1^ effect - but the size of this effect decreased with each successive generation - i.e. negative Generation number^2^ effect). There was no evidence that the effect of Polarity (new vs old pole daughter) changed with increasing generation number (i.e. there was no significant Polarity x Generation number interaction), and the initial number of bacteria in the run had no effect on the doubling times of the focal cells.

| A**) Likelihood-ratio test results** |  |  |  |
| --- | --- | --- | --- |
| **Predictor** | **χ^2^** | **df** | **p** |
| Polarity | 11.222 | 1 | < 0.001 |
| Generation number^1^ | 52.199 | 1 | < 0.001 |
| Generation number^2^ | 32.832 | 1 | < 0.001 |
| Polarity x Generation number^1^ | 0.545 | 1 | 0.460 |
| Polarity x Generation number^2^ | 0.459 | 1 | 0.498 |
| Initial number of bacteria | 0.379 | 1 | 0.538 |
|  |  |  |  |
| **B) Minimum adequate model estimates** |  |  |  |
| **Fixed Effects** | **Estimate** | **SE** | |
| Intercept | 18.842 | 0.828 | |
| Polarity (new pole daughter effect) | -1.390 | 0.414 | |
| Generation number^1^ | 1.065 | 0.144 | |
| Generation number^2^ | -0.032 | 0.005 | |
|  |  |  |  |
| **Random effects:** Channel | ~ 0 | | |
| **Residual variance** | 18.82 | | |

**Figure S1. Position dependent glucose uptake.** Uptake of the fluorescent glucose analogue 2-(*N*-(7-Nitrobenz-2-oxa-1,3-diazol-4-yl)Amino)-2-Deoxyglucose (2-NBDG) in the first (magenta downward triangles) and second bacterium (blue upward triangles) from the top of each dead-end channel of a mother machine filled with an aliquot of a stationary phase *E. coli* culture. Noteworthy, in this experiment bacteria have yet to divide in the mother machine, therefore, the 1^st^ and 2^nd^ bacteria from the top of each dead-end channel are two bacteria randomly drawn from the culture with unknown orientation and pole age (see Fig. 1) rather than old and new pole daughters. The points and error bars are means and standard error of the means of measurements performed on 60 individual bacteria. Paired t-test returned that the amount of 2-NBDG accumulated by the 1^st^ and 2^nd^ bacteria from the top of each dead-end channel after 840s incubation was not statistically different (N=30 pairs, p-value=0.9) The dashed and dotted lines are guides-for-the-eye.


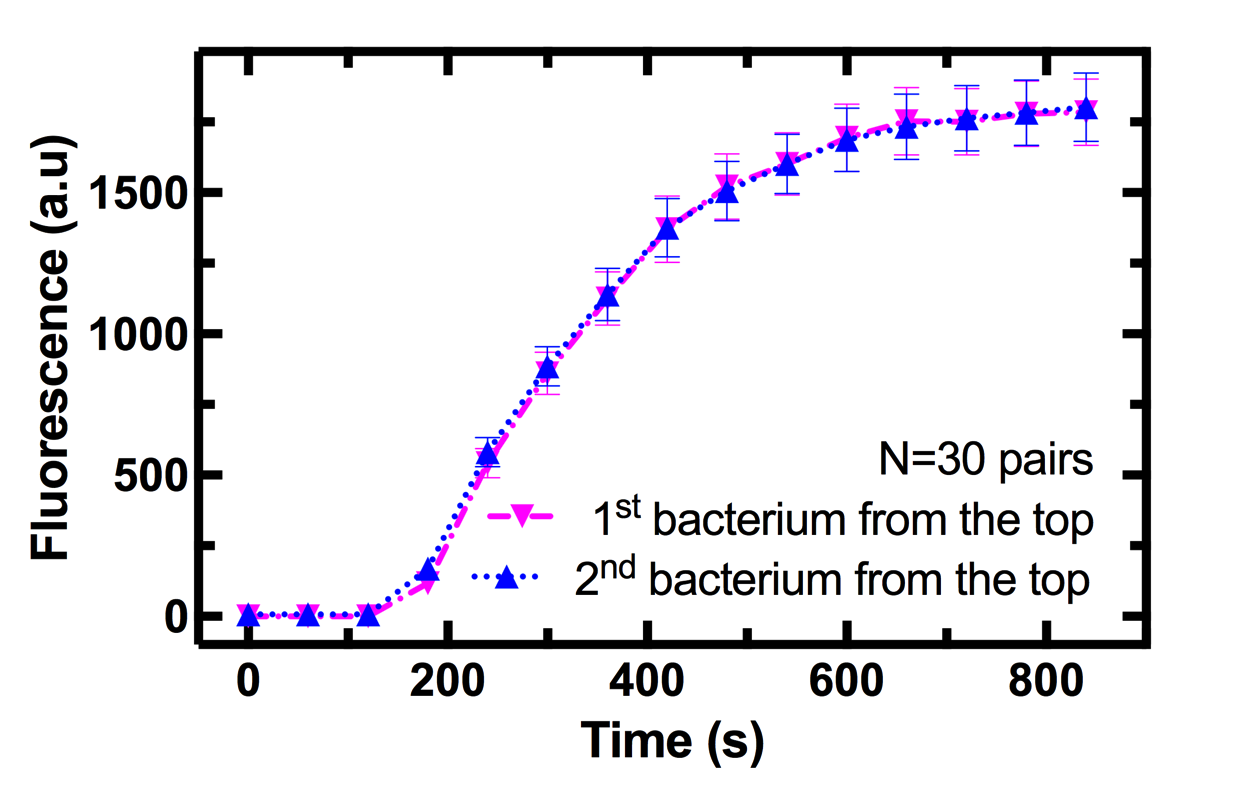


**Figure S2. Ensemble measurements of thioflavin T (ThT) staining.** a) ThT fluorescence intensity as a function of time elapsed after diluting an aliquot of stationary phase *E. coli* in fresh LB or c) M9 with the addition of 0, 5, 20, or 50 µM ThT (black, blue, magenta, and green circles, respectively). Corresponding ThT fluorescence intensity profiles without the addition of *E. coli* to b) LB or d) M9. The data points and error bars are calculated as the mean and standard error of the mean of measurements performed in triplicate. Error bars are hidden behind the data points.


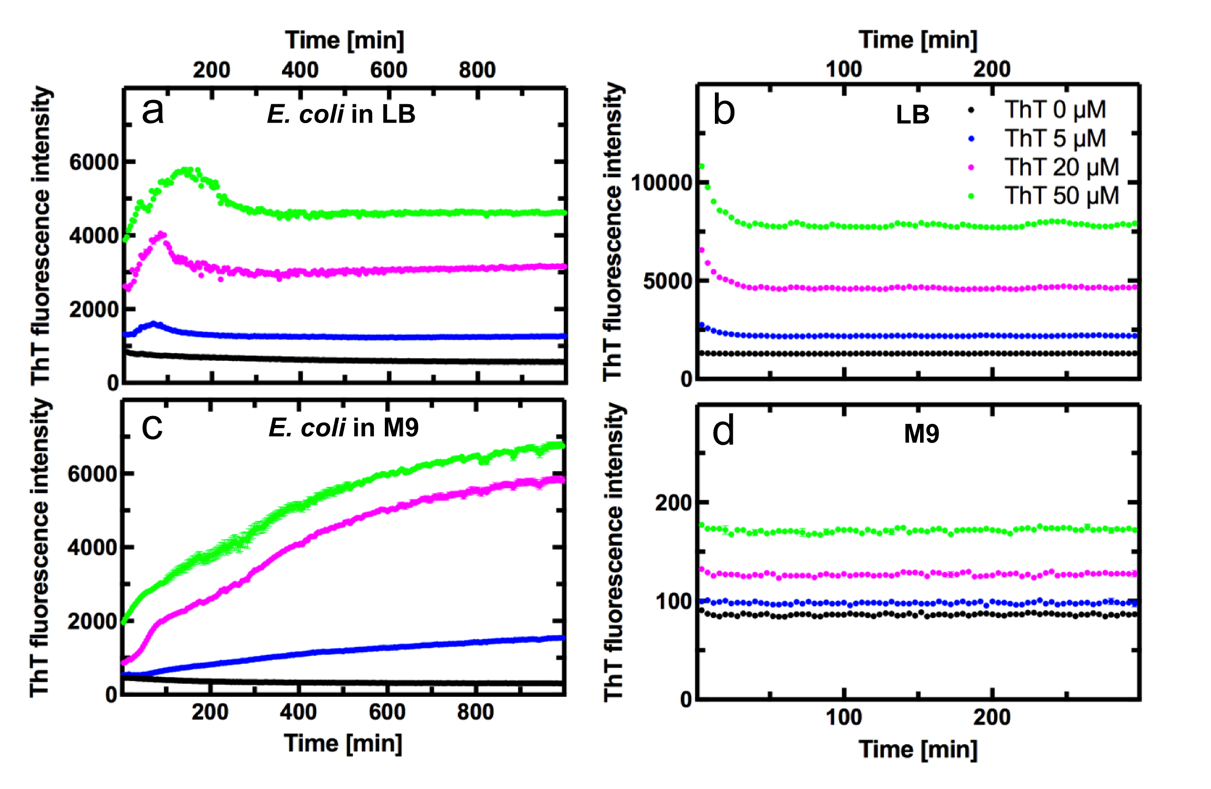

Supplement: Supplementary Material [file rstb20180442supp1.docx]
